# Reply to Navarro-Fernandez, I. Nailing down Clinical Nuances. Comment on “Crotti et al. A Terbinafine Sensitive *Trichophyton indotineae* Strain in Italy: The First Clinical Case of *tinea corporis* and onychomycosis. *J. Fungi* 2023, *9*, 865”

**DOI:** 10.3390/jof10030232

**Published:** 2024-03-21

**Authors:** Silvia Crotti, Deborah Cruciani, Manuela Papini

**Affiliations:** 1 Centro Specialistico Patologie Micotiche, Istituto Zooprofilattico Sperimentale dell’Umbria e delle Marche “Togo Rosati” (IZSUM), Via G. Salvemini 1, 06126 Perugia, Italy; s.crotti@izsum.it; 2 Clinica Dermatologica di Terni, Università degli Studi di Perugia, Piazza Università 1, 06123 Perugia, Italy; manuelapapini@tiscali.it

We have read with interest the comments and observations made regarding the paper “A terbinafine sensitive *Trichophyton indotineae* strain in Italy: the first clinical case of *tinea corporis* and onychomycosis” [1] by Inigo Navarro-Fernandez [2]. We thank you for your favorable comments regarding our work. About the observations regarding the nail lesions, we agree that the images only show onychomadesis of the fourth and fifth fingers of the left hand, an aspect that is not typical of onychomycosis. However, samples collected from the two nails, near the lamina fissure, showed a positive direct examination with KOH for the presence of septate hyphae, and the subsequent culture was equally positive. The positivity of the direct microscopic examination led us to define the lesion as onychomycosis, excluding probable contamination. It is also true that, in the absence of more precise and reliable anamnestic data, any conclusion is difficult.
